# Das ACO-Kurrikulum „Chirurgische Onkologie“ – Voraussetzungen zur Teilnahme, Anmeldung und Ablauf

**DOI:** 10.1007/s00104-023-02016-3

**Published:** 2023-12-28

**Authors:** Tim O. Vilz, Pompiliu Piso, Roger Wahba, Wolfgang E. Thasler, Jörg Kleeff

**Affiliations:** 1 Klinik und Poliklinik für Allgemein‑, Viszeral‑, Thorax- und Gefäßchirurgie, Universitätsklinikum Bonn, Venusberg Campus 1, 53127 Bonn, Deutschland; 2 Klinik für Allgemein- und Viszeralchirurgie, Krankenhaus Barmherzige Brüder Regensburg, Prüfeninger Str. 86, 93049 Regensburg, Deutschland; 3 Klinik für Allgemein‑, Viszeral- und onkologische Chirurgie, Helios Klinikum Berlin Buch, Schwanebecker Chaussee 50, 13125 Berlin, Deutschland; 4 Chair of the UEMS Division of Surgical Oncology, Klinik für Allgemein‑, Viszeral‑, Thorax- und Minimalinvasive Chirurgie, Rotkreuzklinikum München, Nymphenburger Str. 163, 80634 München, Deutschland; 5 Klinik für Viszerale, Gefäß- und Endokrine Chirurgie, Universitätsklinikum Halle (Saale), Ernst-Grube-Straße 40, 06120 Halle, Deutschland

**Keywords:** Chirurgische Onkologie, EBSQ, UEMS, ACO Kurriculum, Surgical oncology, EBSQ, UEMS, ACO curriculum

## Abstract

Bei soliden Malignomen des Gastrointestinaltrakts ist die operative Entfernung ein zentraler Baustein der multimodalen Therapie und oft die einzige Möglichkeit, eine langfristige Heilung zu erreichen. Während für Fachdisziplinen wie Gynäkologie oder Urologie eine onkologische Subspezialisierung existiert, gibt es für die Viszeralchirurgie in Deutschland nichts Vergleichbares, trotz immer komplexer werdender multidisziplinärer Behandlungsstrategien. Durch das ACO-Kurrikulum „Chirurgische Onkologie“ wurde in Kooperation mit der UEMS ein strukturiertes Weiterbildungskonzept geschaffen, das mit der EBSQ-Prüfung „Surgical Oncology“ als Qualitätskontrolle endet. Dies resultiert in einer Verbesserung der chirurgisch-onkologischen Versorgung in Deutschland. Weiterhin erhalten erfolgreiche Absolventen neben dem ACO-Zertifikat eine Urkunde der UEMS und sind Fellow of the European Board of Surgery (FEBS).

## Abstract (en)

For solid malignancies of the gastrointestinal tract, surgical removal is a central pillar of treatment and often the only possibility to achieve a long-term cure. While there are additional qualifications for an oncological subspecialization in other surgical disciplines, such as gynecology or urology nothing comparable exists for visceral surgery in Germany, despite the fact that interdisciplinary cancer treatment strategies are becoming increasingly more complex. The Association of Surgical Oncology (ACO) in cooperation with the European Union of Medical Specialists (UEMS) has created the curriculum for surgical oncology, a structured further education concept, which concludes with the European Board of Surgical Qualification (EBSQ) examination. This results in a standardization and improvement in surgical and oncological treatment in Germany. Furthermore, successful graduates receive an ACO as well as a UEMS certificate and are Fellows of the European Board of Surgery (FEBS).

## Einführung

Bei soliden Tumoren, vor allem bei den Malignomen des Gastrointestinaltrakts, ist die Chirurgie ein zentraler Behandlungspfeiler und oft die einzige Möglichkeit, eine Heilung zu erreichen. Während es in anderen operativen Fächern, wie der Gynäkologie oder der Urologie, Zusatzbezeichnungen hinsichtlich einer onkologischen Spezialisierung gibt, existiert für die Viszeralchirurgie in Deutschland nichts Vergleichbares. Demgegenüber steht eine immer komplexer werdende, multidisziplinäre Krebsmedizin. Damit Chirurg*Innen auch in Zukunft in den Tumorkonferenzen mit den Partnerdisziplinen auf Augenhöhe sinnvolle Entscheidungen zum Wohle ihrer Patient*Innen treffen können, müssen neben den operativen Fähigkeiten auch die Chancen und Risiken einer Chemo‑, Strahlen- und Immuntherapie sowie sonstiger Therapieoptionen realistisch eingeschätzt werden.

Zusätzlich ist aus Registeranalysen bekannt, dass die Qualität in der chirurgischen Onkologie sowie die Fallzahl einen hohen Einfluss auf das kurzfristige, aber auch auf das langfristige onkologische Outcome haben. Dies deutet darauf hin, dass neben einer Zentralisierung der Operationen auch eine zunehmende Spezialisierung im Bereich „Chirurgische Onkologie“ sinnvoll sein könnte [1, 2].

Die European Union of Medical Specialists (UEMS) hat gemeinsam mit der European Society of Surgical Oncology (ESSO) den Europäischen Facharzt „Surgical Oncology“ etabliert. Der Fokus dieser internationalen Ausbildung liegt neben dem Erlangen einer operativen Expertise auch auf theoretischen Grundlagen in Bezug auf allgemeine Tumorbiologie, Grundlagen der medikamentösen sowie strahlentherapeutischen Tumorbehandlung sowie der speziellen Tumortherapie bei soliden Tumoren und endet mit einer Prüfung [3]. Mit dem erfolgreichen Abschluss der Prüfung erlangt man den Titel „Fellow of the European Board of Surgery (FEBS)“.

Die ACO (Assoziation Chirurgische Onkologie) hat sich mit ihrer Gründung der Aufgabe verschrieben, den onkologischen Schwerpunkt in der Chirurgie zu stärken und die onkologische Weiterbildung zu verbessern [4]. Dies erfolgt in Kooperation mit der ESSO und in Anlehnung an das UEMS Global Curriculum for Surgical Oncology. Da sich allerdings die nationalen Begebenheiten in Deutschland hinsichtlich der onkologisch-chirurgischen Weiterbildung teilweise von denen in Europa unterscheiden, wurde das ACO-Kurrikulum „Chirurgische Onkologie“ entwickelt [5].

## Was ist das ACO-Kurrikulum „Chirurgische Onkologie“?

Das ACO-Kurrikulum ist ein mindestens einjähriges Weiterbildungsprogramm im Bereich „Chirurgische Onkologie“. Mit Abschluss des Kurrikulums und erfolgreicher Prüfung des European Board of Surgical Qualification (EBSQ) Surgical Oncology Examens der UEMS erhalten Teilnehmer*Innen die Urkunde zum Europäischen Facharzt „Surgical Oncology“ und sind „Fellow of the European Board of Surgery“ (FEBS).

Zusätzlich wird den Teilnehmer*Innen im Rahmen des nachfolgenden Deutschen Chirurgenkongresses das Zertifikat über eine erfolgreiche Teilnahme am ACO-Kurrikulum im Rahmen der DGAV-Mitgliederversammlung durch den Kongresspräsidenten oder die Kongresspräsidentin verliehen.

## Was sind die Voraussetzungen für eine Teilnahme am ACO-Kurrikulum?

Um am ACO Kurrikulum teilzunehmen, müssen sich die Interessenten direkt bei der ACO bewerben (aco@dgav.de). Diese Bewerbung beinhaltet einen Lebenslauf sowie ein Motivationsschreiben. Die Erstbegutachtung dieser Unterlagen und die Evaluierung der Zulassung zum Kurrikulum erfolgt über die Kerngruppe des Kurrikulums „Chirurgische Onkologie“ der ACO.

Teilnehmer am Kurrikulum sollen in einem Zentrum mit einer vorhandenen DKG-Zertifizierung (z. B. Darmkrebszentrum, Pankreaskrebszentrum, viszeralonkologisches Zentrum etc.) tätig sein.

Pro Jahr stehen zehn Plätze für eine Teilnahme am Kurrikulum zur Verfügung, maßgeblich ist der Zeitpunkt des Eingangs der Bewerbung.

## Was beinhaltet das ACO-Kurrikulum „Chirurgische Onkologie“?

**Figure 1:**
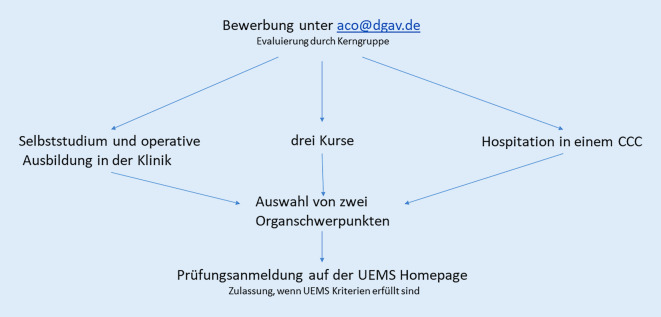

Die einjährige Weiterbildung sollte an einer multidisziplinären Weiterbildungsstätte absolviert werden und setzt sich aus mehreren Blöcken zusammen (Abb. 1):

### Theoretisches Selbststudium

#### A) Allgemeiner Teil

Der allgemeine Teil beinhaltet Grundlagen und beschäftigt sich mit den Nachbardisziplinen. Folgende Themen sind zugehörig:

- Karzinogenese (zelluläre Mechanismen der Karzinogenese)
- Karzinogene (Strahlung, Viren, Chemikalien etc.)
- Epidemiologie von Krebs (epidemiologische Verlaufsdaten, epidemiologische Forschung)
- Früherkennung (Prinzipien, Bias, Risiken, Nutzen, Arten des Screenings)
- Klinische Studien und Forschungsmethoden (Studiendesign, Regularien, statistische Analyse)
- Strahlenbiologie (Wirkmechanismen, Arten der Strahlentherapie, Nebenwirkungen, Dosis)
- Chemotherapie (Chemotherapie, Wirkstoffklassen, Nebenwirkungen, gezielte molekulare Therapieansätze)
- Palliativmedizin (Symptomkontrolle, Patientenverfügung, Patientenwille, sozialmedizinische Unterstützung)
- Psychoonkologie und Gesprächsführung
- Perioperative Medizin

#### B) Spezieller Teil

Der spezielle Teil beinhaltet die organspezifische Therapie. Hier sind tiefgehende Kenntnisse (Screening, Inzidenz, Ätiologie, Genetik, Pathologie, Staging, Diagnosestellung, chirurgische Therapie, neoadjuvante und adjuvante Therapie, Therapie lokal fortgeschrittener und metastasierter Tumoren, Psychoonkologie, Palliation) vor allem bei den Tumorentitäten notwendig, die in Deutschland durch Viszeralchirurgen maßgeblich mitbehandelt werden. Im Einzelnen handelt es sich um:

- Kolorektale Karzinome
- Karzinome des oberen Gastrointestinaltrakts
- Hepatopankreatobiliäre Malignome
- Endokrine/neuroendokrine Neoplasien (Schilddrüse, Nebenschilddrüse, Nebennieren und endokrines Pankreas)
- Sarkome/GIST
- Primäre und sekundäre peritoneale Malignome inklusive Ovarialkarzinom

Da die Prüfung sich am Gegenstandskatalog der UEMS orientiert, sind ebenfalls grundlegende Kenntnisse bezüglich folgender Tumorentitäten erforderlich:

- Mammakarzinom
- Thorakale Malignome
- Hautkrebs und Melanom
- Urologische Malignome
- Gynäkologische Malignome

Allerdings sind prozentual weniger Fragen zu diesen Entitäten zu finden.

Weitere Informationen sowie eine Zusammenfassung der prüfungsrelevanten Themen sind auf der Seite der UEMS einsehbar: https://uemssurg.org/surgicalspecialties/surgical-oncology/.

### Praktisches Selbststudium

Es wird das Erlernen allgemeiner Fähigkeiten wie beispielsweise klinische Untersuchung, Befundung moderner Bildgebung, präoperatives Assessment, perioperative Betreuung, postoperative Betreuung und Rehabilitation, Bedeutung des interdisziplinären Tumorboards, Kommunikationsfähigkeiten etc. vorausgesetzt.

### Drei Operationskurse

Die Teilnehmer*innen sollen zwei ausgesuchte DGAV-Operationsworkshops mit onkologischem Schwerpunkt sowie einen weiteren ESSO-DGAV-, ESSO-, AIO-, DGVS-AGIO- oder DGHO-Kurs innerhalb eines Jahres absolvieren. Eine Auflistung der Kurse ist auf den Internet-Seiten der entsprechenden Fachgesellschaften zu finden.

### Hospitation in einem zertifizierten Comprehensive Cancer Center (CCC) der Deutschen Krebshilfe

Der Teilnehmer*innen am ACO-Kurrikulum sollen eine mindestens einwöchige Hospitation in einem CCC absolvieren. Die Liste der CCCs ist einsehbar unter: www.ccc-netzwerk.de/das-netzwerk/mitglieder.

Sofern die Teilnehmerinnen und Teilnehmer selber an einem CCC arbeiten, empfiehlt sich eine Hospitation in einem anderen CCC.

Die Teilnahme am ACO-Kurrikulum ist kostenfrei.

## In welcher Beziehung stehen das ACO-Kurrikulum und das UEMS-Kurrikulum „Surgical Oncology“

Das ACO-Kurrikulum ergänzt das UEMS-Kurrikulum bezüglich praktischer Aspekte (Operationskurse, einwöchige Hospitation) und fördert explizit die Vernetzung onkologischer Chirurgen in Deutschland. Allerdings kann das ACO-Kurrikulum nur erfolgreich absolviert werden, wenn das EBSQ-Examen der UEMS bestanden wurde. Prinzipiell kann die Prüfung in jedem Land absolviert werden, die mündliche und schriftliche Prüfung sowie die Fallvorstellung erfolgen auf Englisch. Sofern die Prüfung während des Kongresses „Viszeralmedizin“ in Deutschland absolviert wird, erfolgt die Prüfung auf Deutsch.

## Wie wird das ACO-Kurrikulum erfolgreich beendet?

Das ACO-Kurrikulum findet seinen erfolgreichen Abschluss, wenn die EBSQ-Prüfung „Surgical Oncology“ der UEMS bestanden wurde. Die Teilnehmerinnen und Teilnehmer erhalten dann eine Urkunde der UEMS, sind Fellow of the European Board of Surgery (FEBS) und erhalten das Zertifikat der ACO.

## Was sind die Voraussetzungen für eine Teilnahme an der Prüfung „Surgical Oncology“?

Um zur Prüfung zugelassen zu werden und somit das ACO-Kurrikulum erfolgreich abschließen zu können, müssen folgende Kriterien erfüllt sein:

- Facharzt für spezielle Viszeralchirurgie, dann mindestens 4 Jahre Spezialisierung onkologische Chirurgie, davon ein Jahr in einem nationalen oder internationalen onkologischen Zentrum
- Tumorkonferenzteilnahme auch aktiv 1‑mal /Woche
- Chirurgische Ausbildung von erfahrenen Trainern, direktes Feedback, Log-Buch; am Ende der Ausbildung soll der Qualifizierende in seinem Spezialgebiet komplexe Eingriffe selbständig und mit hoher Qualität durchführen können
- Klinische Ausbildung mind. 2‑mal /Woche, diese soll auch Gesprächsführung, z. B. Überbringen schlechter Nachrichten, beinhalten
- Forschung, z. B. Patientenrekrutierung für klinische Studien, Kurse bez. Ethik, Recherchetechniken etc.
- Regelmäßige Treffen mit Mentor bez. Ausbildung
- Teilnehmer am Kurrikulum sollen Zugang zu regelmäßiger hochwertiger Fortbildung erhalten, an nationalen und internationalen onkologischen Kongressen teilnehmen, Zugang zu medizinischer Literatur erhalten; an der Arbeitsstelle sollen die aktuellsten diagnostischen und therapeutischen Möglichkeiten vorhanden sein
- Absolviertes Kurrikulum der ACO über mindestens 1 Jahr ist vorhanden

Neben den Voraussetzungen der ACO müssen auch die Voraussetzungen der UEMS bzw. der ESSO erfüllt sein, welche auf folgender Homepage nochmal dezidiert einsehbar sind:

https://uemssurg.org/surgicalspecialties/surgical-oncology/ebsq-examinations/.

## Kann das ACO-Kurrikulum auch ohne Prüfung absolviert werden?

Grundvoraussetzung für das Zertifikat des einjährigen Kurrikulums ist ein erfolgreiches EBSQ-Examen „Surgical Oncology“. Das EBSQ-Examen kann auch ohne ACO-Kurrikulum abgelegt werden, allerdings erhalten die Absolventinnen und Absolventen auch nur die Europäische Facharzturkunde und nicht das ACO-Zertifikat.

## Wann und wo findet die Prüfung statt?

Die schriftliche Prüfung findet als zweistündige Prüfung online in deutscher Sprache zeitgleich mit der internationalen Prüfung im jeweiligen Frühjahr (Daten siehe https://uemssurg.org/surgicalspecialties/surgical-oncology/ebsq-examinations/) statt.

Nur bei Bestehen der schriftlichen Prüfung erfolgt die Zulassung zur mündlichen Prüfung. Diese findet einmal jährlich zu Beginn der „Viszeralmedizin“ statt. Nach Bestehen der schriftlichen und mündlichen Prüfung wird noch im Rahmen der „Viszeralmedizin“ die Urkunde „Surgical Oncology“ überreicht und der Kandidat oder die Kandidatin dürfen sich „Fellow of the European Board of Surgery“ nennen. Das Zertifikat für einen erfolgreichen Abschluss des ACO-Kurrikulums wird beim folgenden DCK im Rahmen der DGAV-Mitgliederversammlung feierlich überreicht.

## Wie erfolgt die Anmeldung zur Prüfung?

Die Anmeldung erfolgt über die Seite der UEMS, weiterführende Informationen (Deadline zur Anmeldung etc. unter: https://uemssurg.quiz.one/) sind dort ebenfalls zu finden. Die Teilnahme an der Prüfung ist gebührenpflichtig und kostet ab 2024 1000 €.

Die ACO-Kandidat*Innen müssen weiterhin nach Bestehen der schriftlichen Prüfung im Frühjahr unaufgefordert bis zur zum 30. Juni im Prüfungsjahr ihre Fortbildungsnachweise und die Bestätigung über eine einwöchige Hospitation in einem CCC an aco@dgav.de senden, um das Kurrikulum mit dem Abschluss der mündlichen Prüfung während der „Viszeralmedizin“ erfolgreich absolvieren zu können.

## Was sind Prüfungsinhalte?

Die Prüfung besteht aus insgesamt drei Teilen.

### 1. Theoretische Prüfung

Die theoretische Prüfung besteht aus 60 Multiple-Choice-Fragen und dauert 120 min. Jede Frage hat 5 mögliche Antworten, nur eine ist korrekt. Es gibt keine Punktabzüge für falsche Antworten.

In der schriftlichen Prüfung werden neben den viszeralonkologischen Themen, der Sarkomchirurgie und der endokrinen Chirurgie auch grundlegende Fragen zum Mammakarzinom, Melanom etc. gestellt. Nur wer die schriftliche Prüfung erfolgreich absolviert hat, wird zur mündlichen Prüfung zu Beginn der „Viszeralmedizin“ zugelassen.

### 2. Mündliche Prüfung

Bereits im Rahmen der Anmeldung zur Prüfung über die UEMS-Homepage haben sich die Prüflinge auf zwei Organschwerpunkte (z. B. „Kolorektal“ und „Oberer Gastrointestinaltrakt“) festgelegt. Auf diese Organe fokussiert sich die mündliche Prüfung. Die Prüfung gliedert sich in vier 15-minütige Aufgaben, bestehend aus der Diskussion zweier klinischer Fälle sowie zweier wissenschaftlicher Publikationen.

- Diskussion der klinischen Fälle
  Zu den ausgewählten Schwerpunkten werden den Prüflingen zwei Fallbeispiele zugeteilt, in denen mit den Prüfern das Vorgehen von der Erstvorstellung bis zur Nachsorge und Prognose diskutiert wird.
- Wissenschaftliche Publikationen
  Die Prüflinge erhalten zwei wissenschaftliche Publikationen von bedeutenden klinischen Studien im Fachgebiet chirurgische Onkologie mit thematischem Bezug zu den vorher gewählten Organschwerpunkten. Nach 2 h Vorbereitungszeit werden die beiden Manuskripte mit den Prüfern diskutiert und Fragen hinsichtlich des Ziels der Studie, des Studiendesigns, der Ergebnisse, Stärken und Schwächen beantwortet.

Die gesamte Prüfung erfolgt in deutscher Sprache. Die Prüfer*Innen sind erfahrene deutschsprachige chirurgische Onkolog*Innen und haben alle erfolgreich die EBSQ-Prüfung „Surgical Oncology“ in der Vergangenheit absolviert (Tab. 1).

**Table 1:** 

Prof. Dr. med. Stefan Farkas	Wiesbaden
PD Dr. med. Gabriel Glockzin	München
Prof. Dr. med. Axel Kleespies	Dachau
Prof. Dr. med. Jörg Pelz	Hildesheim
Prof. Dr. med. Daniel Perez	Hamburg
Prof. Dr. med. Pompiliu Piso	Regensburg
Prof. Dr. med. Beate Rau	Berlin
PD Dr. med. Stefan Stättner	Vöcklabruck
Prof. Dr. med. Wolfgang Thasler	München
Prof. Dr. med. Tim Vilz	Bonn
Prof. Dr. med. Roger Wahba	Berlin

## Wie bereite ich mich auf die Prüfung vor?

Zur Prüfungsvorbereitung empfiehlt sich eine Orientierung am ESSO Core Curriculum (Curriculum and Syllabus – UEMS Section of Surgery [uemssurg.org]). Weiterhin hilfreich sind aktuelle Leitlinien der AWMF und die ESMO Guidelines (ESMO | ESMO) sowie die Kenntnisse der wichtigsten chirurgisch-onkologischen Studien aus den letzten Jahren.

Folgende Lehrbücher sind hilfreich:

1. *Evidenzbasierte Viszeralchirurgie maligner Erkrankungen: Leitlinien und Studienlage*
  Christoph-Thomas Germer, Tobias Keck, Reinhart T. Grundmann
2. *Surgical Oncology: Theory and Multidisciplinary Practice*, 2nd Edition
  Graeme J. Poston, Lynda Wyld, Riccardo A. Audisio

## Fazit

Mit dem Kurrikulum ist ein deutschsprachiges Weiterbildungsprogramm verfügbar, das eine systematische Ausbildung vom onkologisch tätigen Chirurgen zum chirurgischen Onkologen ermöglicht. Durch den Aufbau des ACO-Kurrikulums mit der abschließenden EBSQ-Prüfung erfolgen eine Ausbildung mit Prüfung und somit auch eine Qualitätskontrolle/Zertifizierung. Hierdurch wird eine Standardisierung und Verbesserung der chirurgisch-onkologischen Versorgung erreicht. Dies macht sich auch in den Tumorkonferenzen bemerkbar, da die Chirurgie aufgrund der breiten, interdisziplinären Ausbildung und dem Blick über den chirurgischen Tellerrand als gleichberechtigter Partner*In und nicht als nur auf Zuruf tätiger Operateur*In angesehen wird. Deutschland beschreitet mit der DGAV hier auch eine Vorreiterrolle in der Implementierung der Europäischen Facharztqualifikation auf nationaler Ebene. Unter dem Dach der UEMS werden mittlerweile auch die Prüfungen in italienischer, französischer und spanischer Sprache etabliert und abgehalten.

Zum jetzigen Zeitpunkt existiert weder ein Facharzt noch eine Zusatzbezeichnung für chirurgische Onkologie. Setzt man sich allerdings genauer mit dem Europe’s Beating Cancer Plan der ESSO auseinander, ist dort eine entsprechende Zusatzqualifikation mittelfristig als Ziel ausgerufen [6].
